# An Improved Flexible Telemetry System to Autonomously Monitor Sub-Bandage Pressure and Wound Moisture

**DOI:** 10.3390/s141121770

**Published:** 2014-11-18

**Authors:** Nasir Mehmood, Alex Hariz, Sue Templeton, Nicolas H. Voelcker

**Affiliations:** 1 School of Engineering, University of South Australia, Adelaide, SA 5001, Australia; E-Mail: alex.hariz@unisa.edu.au; 2 Royal District Nursing Service, Adelaide, SA 5035, Australia; E-Mail: sue.templeton@rdns.org.au; 3 Mawson Institute, University of South Australia, Adelaide, SA 5001, Australia; E-Mail: nico.voelcker@unisa.edu.au

**Keywords:** chronic wound management, wound sensing and monitoring, telemetric sensing, wound diagnostics, mobile-based biomedical systems

## Abstract

This paper presents the development of an improved mobile-based telemetric dual mode sensing system to monitor pressure and moisture levels in compression bandages and dressings used for chronic wound management. The system is fabricated on a 0.2 mm thick flexible printed circuit material, and is capable of sensing pressure and moisture at two locations simultaneously within a compression bandage and wound dressing. The sensors are calibrated to sense both parameters accurately, and the data are then transmitted wirelessly to a receiver connected to a mobile device. An error-correction algorithm is developed to compensate the degradation in measurement quality due to battery power drop over time. An Android application is also implemented to automatically receive, process, and display the sensed wound parameters. The performance of the sensing system is first validated on a mannequin limb using a compression bandage and wound dressings, and then tested on a healthy volunteer to acquire real-time performance parameters. The results obtained here suggest that this dual mode sensor can perform reliably when placed on a human limb.

## 1. Introduction

The treatment of chronic wounds such as venous leg and diabetic ulcers, has emerged as one of the greatest scientific as well as financial challenges for the global medical community [1]. The average cost per person for a 2-year diabetic foot ulcer treatment in the USA was estimated at $27,987 in 1999 [2]. A current estimate shows the economic cost of woundcare activities in the world is distributed as 15%–20% materials, 30%–35% nursing time, and more than 50% as hospitalization time [3]. It was estimated in 1991 that the prevalence of leg ulcers only within the USA was between 0.5% and 1.5% with an annual cost of nearly US$1 billion [4]. In the UK, during 2001, chronic wounds were a major cause of morbidity, affecting more than 1% of population and with treatment cost of at least £1 billion [5]. During 2006–2007, chronic wounds were affecting 3–6 million people in the USA with a total cost of treatment estimated at more than $3 billion annually [6,7]. In 2012, the number of people suffering from chronic wounds was 7 million, and the cost for their treatment was estimated at almost $25 billion annually [8]. Irrespective of the financial burden, chronic wounds have significant social and economic implications in the form of increased hospitalisation rates, and reduced quality of life for patients.

So far, the most effective and economical treatment of wounds is to cover them with a suitable dressing in order to provide the wound a conducive environment to heal [9]. It has been proven that moist dressings, such as hydrocolloids, hydrogels and foams, are helpful in improving the healing rate as well as in reducing the pain associated with chronic wounds [9]. For certain chronic wounds (e.g., venous leg ulcers), compression therapy is provided as a first-line treatment using bandages or stockings [10,11], a technique used in clinical practice for many centuries [12,13]. Compression bandages are designed to produce a sub-bandage pressure up to 60 mmHg at the ankle, which is regarded as ‘extra-high’ pressure, while the recommended high sub-bandage pressure value for treatment of venous leg ulcers is 40 mmHg at the ankle [14,15]. Depending on the applied pressure range and the type of bandage used, the sub-bandage pressure may vary significantly during the physical movement of the patient, thus affecting the healing rate [16]. In current clinical practice, these and other critical wound parameters (identified in [17]) are not monitored to track the efficacy of treatment.

An earlier method to monitor sub-bandage pressure using a rigid fontanometer sensor along with external electronics was proposed by Wertheim *et al.* in 1999 [12]. McColl *et al.* [18] developed an impedance-based moisture sensing system for wounds. Following this, Ohmedics^©^ (Glasgow, UK) developed a clinically-proven moisture monitoring device called WoundSense^®^ [19]. However, this device was not designed to stay within the dressing for wound moisture monitoring. Khaburi *et al.* [20] reported a force sensor-based pressure-mapping bandage prototype to measure pressure at various points on a leg mannequin. However, the wired connections between the sensors and the processor inhibit the practical utilization of the system. Nevertheless, miniaturized pressure sensors have been designed and used in other biomedical applications including intracranial pressure [21], intraocular pressure [22], spinal plates pressure [23], and for general *in vivo* applications [24]. Whilst researchers have proposed integrated monitoring systems using wireless data transmission [25,26], those devices have neither been tested in a wound environment nor was their wireless transmission range (3.5 cm and 4 cm) sufficient for any practical use.

In our earlier review article, we have identified the research gap in the utilization of sensor technology for chronic wound management [27]. We have also demonstrated the first prototype telemetric sensing system for chronic wound monitoring [28]. The system was capable of measuring and transmitting real-time information on temperature, moisture, and sub-bandage pressure from under the bandage or within the wound dressing at programmable transmission intervals. The sensing system was fabricated on flexible printed circuit material, while the sensors were micro-sized and flexible; thus making the system minimally invasive to wounds and the human body. The receiver was portable with the capability to receive data accurately within a distance of 4–5 m. The system was tested on a human volunteer using various compression bandages and moisture-retentive wound dressings applied by an experienced wound management nurse. The results from these trials confirmed the practical utility of this system for wound monitoring and for other biomedical applications. However, the size of the system was too large and the performance too unreliable [29].

In this paper, we demonstrate an improved wound monitoring system fabricated on a 0.2 mm flexible printed circuit material. The sensing system is smaller in size, and is fit for clinical purpose. The following sections explain the design, operation, and experimental results of the proposed system.

## 2. Sensing System Design and Operation

**Figure 1.**
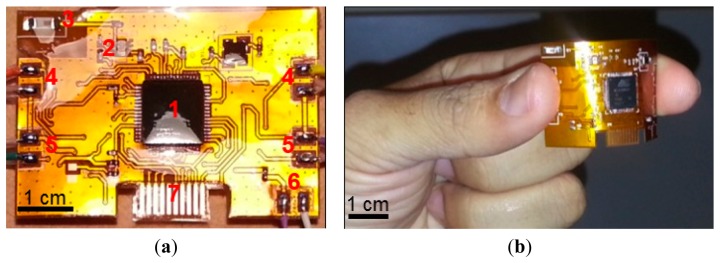

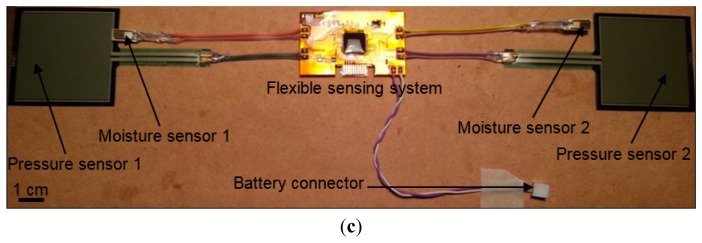
(**a**) The improved wound sensing system with its various parts annotated as (1) RF transmitter, (2) RF balun, (3) Antenna, (4) Interface for moisture sensor, (5) Interface for pressure sensor, (6) Interface for battery, and (7) Chip programming interface. The sensing system is coated with a layer of biocompatible material polydimethylsiloxane (PDMS); (**b**) the flexible telemetric sensing system shown without sensors; (**c**) the complete wound sensing system with all the sensors interfaced.

The telemetric sensing system (Figure 1) is composed of two pressure sensors and two moisture sensors interfaced to an active radio transmitter through specialized interface circuits. With the use of twin pressure and moisture sensors, the system is able to measure pressure and moisture at two different locations simultaneously within a wound dressing or bandage.

The system allows sub-bandage pressure at ankle and calf muscle to be measured, while the moisture level in a wound dressing could also be measured by inserting one sensor into a dressing placed over the wound site and the other over normal skin for reference.

All the sensors are interfaced to an active radio frequency (RF) ATMega128RFA1 ZigBee^®^ transmitter, through dedicated interface circuits mounted on the rear of the sensing system. This radio device is chosen because it provides a single-chip solution for data acquisition, conversion, storage, and transmission. The sensing system is powered through a 6.0 V 165 mAh alkaline battery.

### 2.1. Sensors and Interfacing

The sensing system uses commercially available pressure and moisture sensors deemed suitable for placement under a wound dressing or bandage. A majority of available sensors do not qualify for this particular application because of their large size, invasive structure, complex principle of measurement, and the need for additional on-board circuit components.

For wound monitoring application, the sensors need to be biocompatible and non-invasive to the human body, as the sensors would be placed within a wound dressing or under a compression bandage over a human limb [30]. Hence, we chose the FSR406 (Interlink Electronics®, Camarillo, CA, USA) flexible force sensor for sub-bandage pressure, and a HCZ-D5 (Multicomp Farnell®, Leeds, UK) 2-wire passive sensor for moisture measurement. The dimensions of these sensors are 38 mm × 38 mm × 0.5 mm, and 10 mm × 5 mm × 0.5 mm, respectively. The pressure sensor was calibrated and characterized for the pressure range 0–60 mmHg, while the moisture sensor was calibrated and characterized for 0%–100% RH.

**Figure 2.**
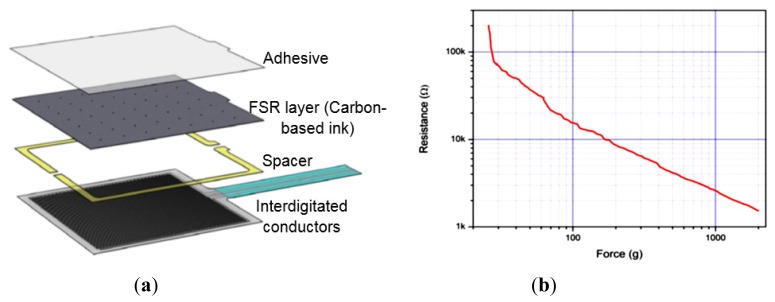
(**a**) Basic architecture of the FSR406 pressure sensor used in the sensing system; (**b**) a typical Force *vs.* Resistance curve for the FSR series sensors. Figures reused from the manufacturer's datasheet [31] with permission from Interlink Electronics®.

The chosen pressure and moisture sensors are analog passive resistive sensors *i.e.*, a change in the applied quantity (pressure or moisture) results in a change in resistance or impedance between two output terminals of the sensors. The FSR406 sensor consists of two interdigitated electrically-conductive traces placed under a thin conductive polymer sheet coated with carbon-based ink [31] (Figure 2a). When pressed with a force, the ink shorts portions of conductive fingers together with a resistance that depends on the quantity of applied force. An increase in force applied on the surface of the sensor shorts more traces together and thus decreases the resistance between its terminals (Figure 2b). Calculations derived from the datasheet of FSR406 sensor show that small variations in operating temperature (e.g., 25 ± 5 °C) may produce very small changes in resistance (±1.25%).

**Figure 3.**
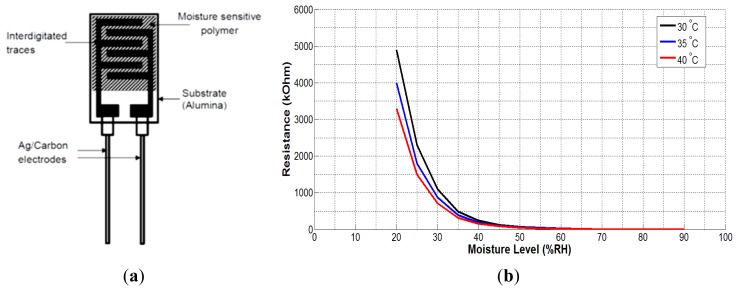
(**a**) Composition of the HCZ-D5 moisture sensor; (**b**) plot of sensor's impedance *vs.* moisture level for the desired temperature range 30–40 °C based on the data supplied by the manufacturer.

The moisture sensor (HCZ-D5) consists of interdigitated conducting silver-carbon electrodes placed over a very thin layer of moisture-sensitive material (polymer). The resistance of the polymer is almost infinite in complete dry conditions. Moisture on the surface of the sensor decreases the resistance of the polymer material connecting the two electrodes. The datasheet of the sensor provides values of resistance *vs.* moisture level for the temperature range 5–60 °C. However, for the wound sensing system, the temperature range of 30–40 °C is of particular interest as the skin temperature normally remains within this range. Figure 3b shows the graphs of impedance changes with moisture level for the temperature range of interest. The graph shows a non-linear variation of sensor's impedance with moisture level. It is also evident from the graph that temperature variations have significant effects on sensor's impedance for the moisture range 20%–45% RH after that the effect is minimal. As the skin temperature varies between 32 and 37 °C, the graph with 35 °C was deemed as most appropriate for calibration of the moisture sensor.

**Figure 4.**
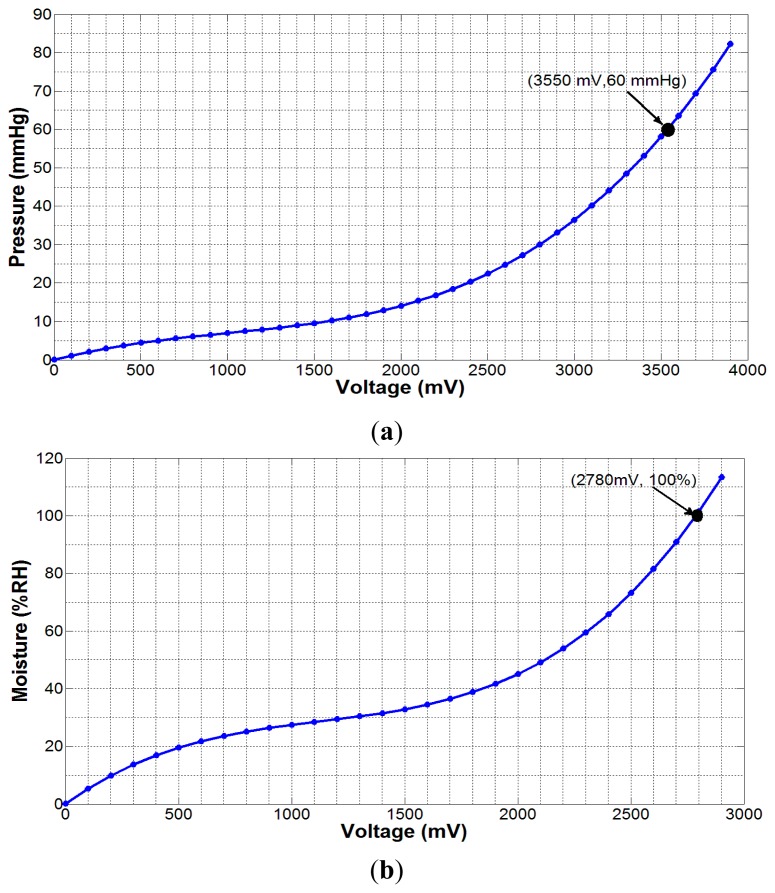
(**a**) Experimental pressure data fitted using Equation (1); (**b**) experimental moisture data fitted using Equation (2). The dot point in both the graphs indicates the maximum expected value of the respective measured parameter.

In order to measure the quantities from these sensors, we need to transform the change in resistance/impedance into a change in voltage level because the forthcoming operations in the telemetry device are based on voltage level measurement only. For this purpose, we used LM358 (Texas Instruments, Dallas, TX, USA) full-swing operational amplifier (op-amp) along with other required circuit components. Using dedicated experimental setups involving a pneumatic pressure meter Kikuhime® (Advancis Medical, Nottinghamshire, UK), a generic moisture probe, an elastic compression bandage (AMS Bi-Flex^®^, Canning Vale, WA, Australia), a mannequin leg, a data acquisition board and a curve-fitting software, the following mathematical expressions for output voltages (in mV) were obtained for the pressure and moisture sensors, respectively:

(1) $$P\left(mmHg\right)=p_{1}{V_{\text{out}}}^{3}+p_{2}{V_{\text{out}}}^{2}+p_{3}V_{\text{out}}+p_{4}$$

(2) $$M\left(\%\right)=m_{1}{V_{\text{out}}}^{3}+m_{2}{V_{\text{out}}}^{2}+m_{3}V_{\text{out}}$$

where, p_1_ = 2.533 × 10^−9^, p_2_ = −7.553 × 10^−6^, p_3_ = 0.012, p_4_ = −0.00921, and *m_1_* = 1.228 × 10^−8^, *m_2_* = −4.178 × 10^−5^, *m_3_* = 0.05692. Both equations represent nonlinear relationships between the applied quantities and respective output voltages, as shown in Figure 4.

The analog voltage at the output of LM358 op-amp represents the change in the applied quantity to be measured. The voltage is converted into digital using the in-built 10-bit analog to digital converter (ADC) of the radio transmitter. The ADC input range is reduced by using voltage divider circuits in both interface circuits. The digital data is then transmitted over-the-air to the receiving mobile device. On the processing side, the original information is correctly retrieved by multiplying the received data with the voltage division factor used in the interface circuits.

### 2.2. Telemetric Operation of Integrated System

**Figure 5.**
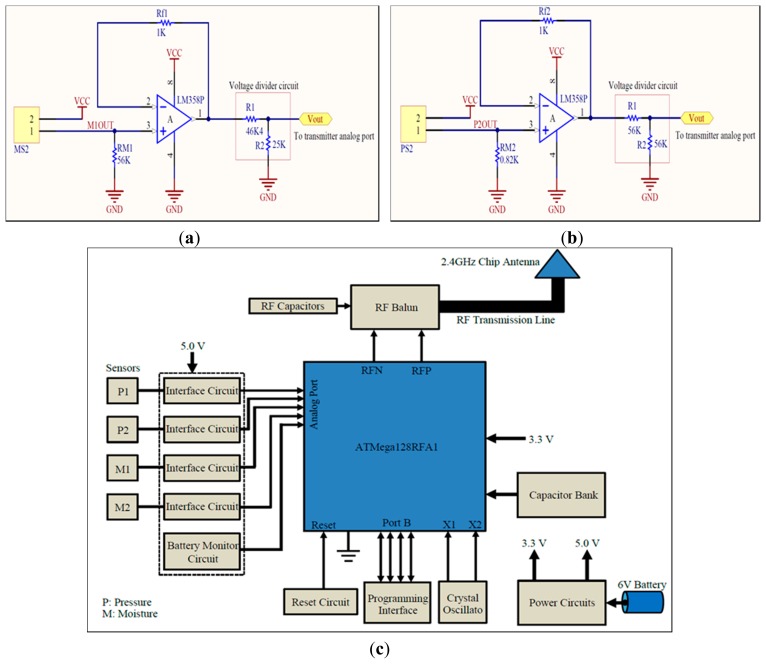
(**a**) Schematic of interface circuit for the moisture sensor HCZ-D5; (**b**) schematic of interface circuit for the pressure sensor FSR406; (**c**) block-level circuit schematic for RF telemetry device and its external interfaces.

The sensors, interface circuits, radio transmitter, and associated components were integrated on a 0.2 mm thick flexible printed circuit material. The circuit schematics for the sensors' interfaces and RF telemetry are shown in Figure 5a–c. The smallest possible components were used to keep the size of the sensing system to a minimum. The sensors' analog signals were applied to analog port F (PF0-3) of the RF transmitter. Highly accurate low drop-out 250 mA, 5.0 V and 400 mA 3.3 V voltage regulators (TPS73250DBVT and TPS73633DBVT from Texas Instruments) were used to supply stable voltages. The system was powered through a 6.0 V 165 mAh 4LR44 standard alkaline battery. The RF transmitter also required an external 16.0 MHz crystal oscillator, 2.45 GHz antenna, and impedance matching network (*i.e.*, RF balun). A highly accurate crystal oscillator (Model: NX3225SA-16MHZ) was used to produce stable clock signals. A 500 mW chip antenna (P/N 2450AT18A100 Johanson Technology^®^, Camarillo, CA, USA) was used along with an RF balun chip (P/N 2450FB15L0001 Johanson Technology^®^) for proper RF transmission (Figure 5c). The length and width of the copper track between the RF balun and the chip antenna were carefully chosen to balance impedance between the two RF components.

The nominal current consumption of the sensing system was measured as 15.7 mA, while the maximum current consumption was 26.0 mA. Hence, the average current consumption was almost 20 mA. Using a 165 mAh battery, the system can be operated continuously for 8.25 h. This means that the system can take approximately 495 measurements if operated once a minute. If measurement interval is prolonged to 10 min, the system could last for almost 34 days. However, the actual life time may be reduced due to leakage power consumption of integrated circuits which is normally in the order of a few micro amperes (μA).

The IEEE 802.15.4 (ZigBee) wireless standard for data transmission and reception was used because of its long range and simple radio operations. The RF chip was programmed to capture, digitize, and transmit the sensors' data at 5 s intervals for experimental purposes only. This interval could be prolonged to multiple minutes or hours as desired, in order to save battery power. The program starts with defining required program libraries, frame buffer, and some variables for temporary storage of information. The transceiver is run through a sequence of states, and then its transmission parameters are defined including channel frequency, data rate, output power *etc*. Next, in an endless loop, the chip is directed to capture each analog signal from the sensors in sequence, perform ADC operation on them sequentially, and then store the result into the frame buffer for transmission. Once the complete packet of information is transmitted in the air, a frame-transmission-end signal is generated by the transmitter.

## 3. Processing of the Received Information

The transmitted information was received by a matched RF receiver, and was then transferred to a mobile device for processing and display. This module received power through the USB interface of the mobile device (a Google Nexus tablet in this work). An Android application (App) was developed for automatic data acquisition, processing, and display in various formats. The App displayed the transmitter device identification, received-signal strength, pressure values, moisture values, and the battery voltage of the sensing system. The App was initialized by setting the lower and the upper limits for the moisture and pressure measurements. It provided visual and audio alerts to the user in abnormal conditions. The measured values within the defined limits were displayed in green colour, while those smaller than the lower limits appear in orange and those higher than the upper limits appear in red colour. The App also displayed a text message on the screen if the sensed battery voltage dropped below a defined threshold (e.g., 3.75 V). The measured data acquired by the App was saved in the internal memory with the time of acquisition for subsequent analysis by the healthcare professionals. The App also displayed the saved data in an interactive graphical form. These features were intended to help healthcare professionals in quick analysis of measured parameters for better evaluation and effective treatment plans.

The measurement process of wound parameters may be affected by external environmental factors such as temperature variations, supply voltage variations, and ambient pressure. The measured values of pressure and moisture might deviate from their actual values under the influence of these factors. This would eventually affect the decision-making process by a healthcare professional. For instance, if the displayed pressure value is lower than it actually is, the patient or clinician may wrongly tighten the bandage to increase the pressure. A similar situation may also arise for other miscalculated wound parameters. This requires the development of an error-correction algorithm that could adjust the values of measured parameters in accordance with the changing external factors.

Moisture measurements are not dependent on ambient pressure; however, the other two factors may affect those measurements. Moisture sensor has already been calibrated for 35 °C which is very close to the average temperature of the skin where the moisture sensor would operate. The sensor calibration is carried out at normal atmospheric pressure. Given that the pressure sensor is based on piezoresistive mechanism, any drift resulting from variations to ambient pressure is negligibly small and ranges at the noise level, where it poses no ambiguity to the measured signal. Moreover, each pressure measurement starts with a 0.0 mmHg value which cancels the effect of ambient pressure if any.

For the pressure sensor FSR406, the impedance between its terminals is a function of temperature, however, the datasheet of the sensor does not describe the temperature co-efficient. To quantify the effects of temperature variations on sub-bandage pressure measurements, we performed an experiment using a calibrated temperature-controlled oven. The sensing system was powered through a fixed 6.0 V supply voltage. The pressure sensor was placed around a hard cylindrical object with 30 mm diameter, and then a constant pressure (∼38 mmHg) was applied over it through a compression bandage at 25 °C. The sensing system along with the loaded pressure sensor was placed inside the oven. The temperature of the oven was monitored through a calibrated temperature probe connected with a high-precision multimeter (Finest^®^ 707 True RMS Multimeter, Sosa-Gu, South Korea). While the loaded pressure sensor was inside the oven, the temperature was increased gradually from 25 °C to 40 °C at a rate of 0.05 °C/s. Pressure readings were recorded through the App after every 1 °C rise in temperature. At the end of the experiment, average error in measurements was calculated with reference to initial value at 25 °C. The measured average error was 0.46 mmHg or 1.21% which might be deemed negligible.

**Figure 6.**
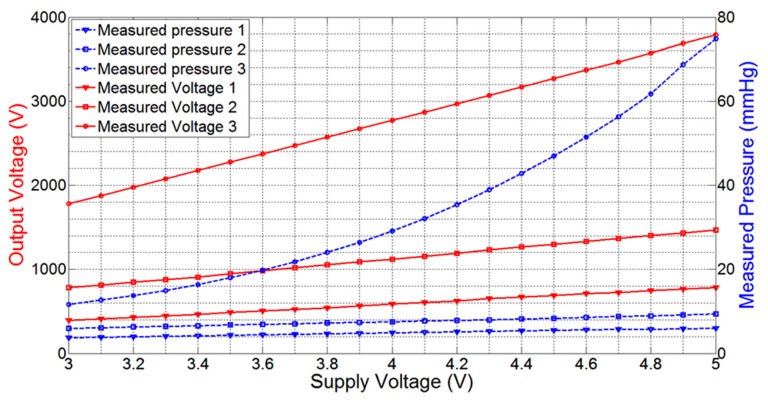
Graphical plot of measured output voltages of LM358 op-amp *versus* supply voltage, and resulting measured pressure values for three different constant pressures on the pressure sensor.

As the telemetric sensing system is powered up through an external battery with limited power, the real-time measurements of pressure and moisture are expected to deteriorate with a drop in battery power. In order to determine the effect of battery power loss on the measurement process, we performed an experiment with the pressure sensor FSR406 placed on a mannequin leg powered through a calibrated and precise power supply 829G (RFL Industries Inc., Boonton Township, NJ, USA). The pressure sensor was attached to its dedicated interface circuit. An elastic compression bandage was applied over the pressure sensor to create a fixed pressure. A stable 5.0 V power was supplied to the sensor module. After validating the fixed pressure on the sensor using a commercial clinical-grade pneumatic pressure meter HPM-KH-01 Kikuhime^®^ (Advancis Medical, Nottinghamshire, UK), the output voltage of the LM358 op-amp was measured for every 100 mV drop in supply voltage until the supply voltage was reduced to 3.0 V, the minimum voltage required to operate the op-amp. The experiment was repeated for two other higher pressure values, and the resulting drops in output voltage as well as in measured pressure were recorded. It was observed that the average output voltage drop was 19.0 mV, 33.0 mV, and 96.0 mV per 100 mV drop in supply voltage for the three applied bandage pressures, respectively. The drop in measured output voltages of LM358 op-amp and the resulting pressure measurements using Equation (1) are plotted in Figure 6. This graph shows that the voltage drop increases with the applied pressure. At high sub-bandage pressure (pressure 3), the slope of output voltage line (voltage 3) is higher than those for other two low pressure values. The graph of ‘pressure 3’ is also non-linear as compared to other graphs because a comparatively higher voltage in Equation (1) gives more weight to higher-order polynomials and hence increases non-linearity.

In order to obtain a precise mathematical expression for the output voltage drop per 100 mV drop in supply voltage, we used a MATLAB curve-fitting tool to obtain the following expressions of first degree polynomial, second degree polynomial, and Gaussian form, respectively:

(3) $$V_{\text{drop}}=0.02598\times V_{\text{out}}-2.974$$

(4) $$V_{\text{drop}}=2.281\times 10^{-6}\times V_{\text{out}}^{2}+0.01516\times V_{\text{out}}+5.789$$

(5) $$V_{\text{drop}}=104.7\times e^{-\left(\frac{V_{\text{out}}-4668}{2976}\right)}$$

where, *V_drop_* and *V_out_* are both expressed in mV.

**Figure 7.**
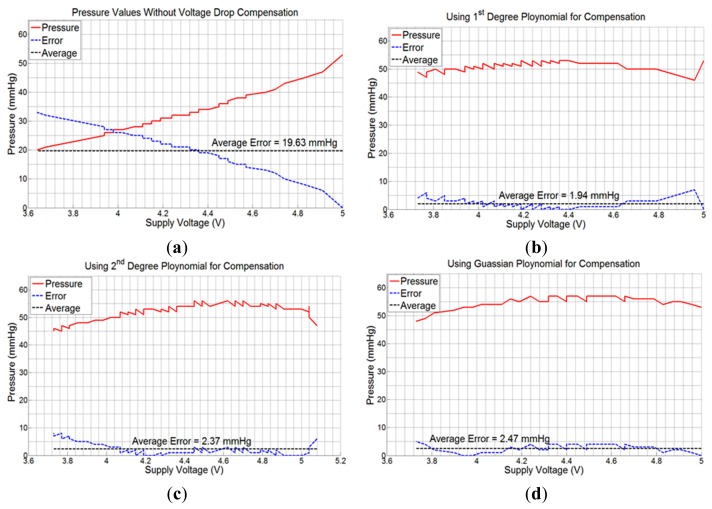
Graphical plots of measured pressure, error, and average error; (**a**) without using any compensation algorithm; (**b**) using 1st degree polynomial in Equation (3); (**c**) using 2nd degree polynomial in Equation (4); (**d**) using Gaussian exponential function in Equation (5).

Next, experiments were performed with the sensing system powered with a 6.0 V battery, and placed on the mannequin leg with the compression bandage applied. Consecutive values of sub-bandage pressure and battery voltage were recorded in the mobile device with 5 s measurement interval. The average error (calculated by taking arithmetic average of all individual errors) produced was 19.63 mmHg or 37.03% with respect to original pressure value of 53 mmHg at 5.0 V (Figure 7a).

Measurements were then taken by repeating the same experiment using voltage drop compensation formulas in Equations (3)–(5), one at a time. The average errors obtained were 1.94 mmHg (3.66%), 2.37 mmHg (4.47%), and 2.47 mmHg (4.66%) for the three equations, respectively (Figure 7b–d). All the experiments were performed using the same experimental setup and with a new battery each time. As is evident from Figure 7b, the 1st degree voltage compensation formula (Equation (3)) provided the best error compensation characteristics. Hence, this formula was used in the App to compensate the received data for battery voltage drop.

## 4. Experiments and Results

**Figure 8.**
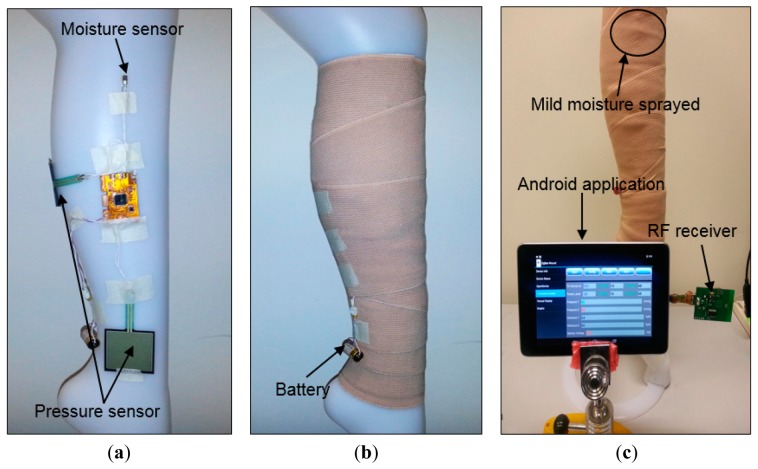
(**a**) Placement of the sensing system, pressure, and moisture sensors prior to application of bandage; (**b**) flexible pressure bandage wrapped over the sensing system; (**c**) measurements using the receiver attached to the mobile device running the developed App for data acquisition, processing, and display.

The improved sensing system was tested on a mannequin leg (Figure 8) using an elastic compression bandage AMS Bi-Flex®. The mannequin limb mimics the curved morphology of a human body part, so it was used to emulate realistic measurement scenarios. The sensing system was placed conformal at the center of the leg. One pressure sensor was placed near the ankle, while the other was placed on the calf section. A clinical grade pneumatic pressure meter (Kikuhime^®^) was used to measure and verify the applied sub-bandage pressure. The moisture sensor was inserted into a foam dressing used to absorb wound exudate (Figure 8a) (only one moisture sensor was used). The compression bandage was applied over the system with a reasonable tightness (Figure 8b), and the sensing system was powered up with the battery. Approximately 3 mL distilled water was sprayed over the bandage portion close to the moisture sensor. The data was acquired through the receiver module attached to the mobile device (Figure 8c).

**Figure 9.**
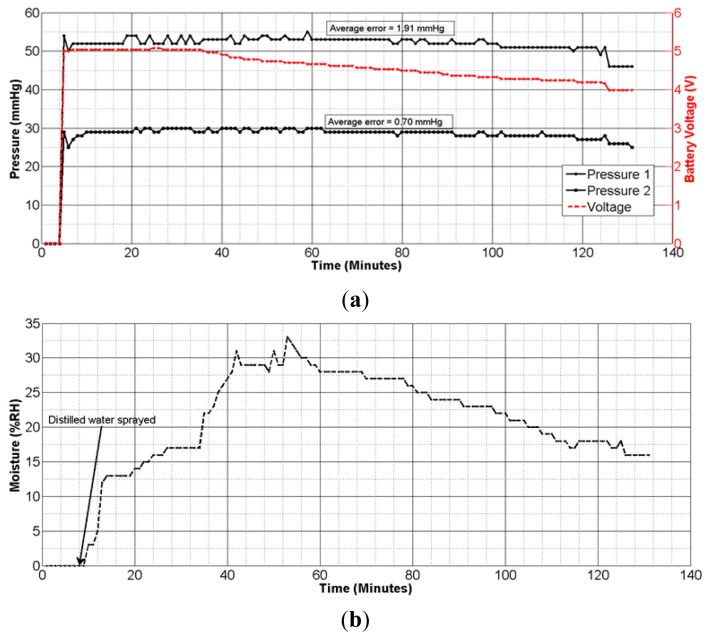
(**a**) Graphical plot of pressure measurements on a mannequin leg over time; (**b**) graphical plot of moisture measurements on a mannequin leg over time.

**Figure 10.**
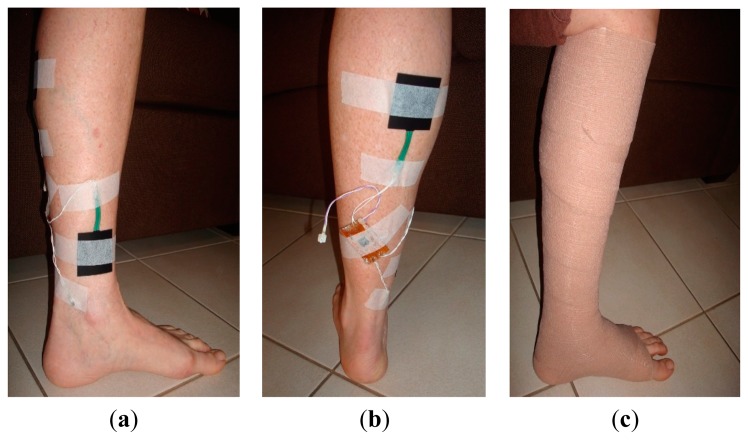
Experiments on a healthy human volunteer; (**a**) placement of one pressure sensor at above the ankle; (**b**) placement of the second pressure sensor at calf level. The telemetric sensing system was also attached on the limb with tape; (**c**) pressure sensors and the telemetry device hidden under the bandage.

The pressure measurement results (Figure 9a) showed an average error of ±1.91 mmHg for pressure measurements at the calf, and ±0.70 mmHg for those at ankle level. These results confirm the accuracy and reliability of measurements with the dropping battery voltage level, attributed to the use of voltage compensation algorithm discussed in the previous section. The moisture measurement results (Figure 9b) indicated a gradual rise and fall in moisture levels consistent with the environment near the moisture sensor. The performance of the sensing system was also tested with experiments on a healthy human volunteer (wound specialist nurse) using 4-layer (Profore^®^, Smith & Nephew Pty Ltd, Kent Town, SA, Australia) and 2-layer (Coban™ 2, 3M Australia, North Ryde, NSW, Australia) compression bandages. To measure sub-bandage pressure at two different locations, one sensor was placed above the ankle (Figure 10a), while the other was placed on the calf muscle (Figure 10b). The complete system was then covered with the bandage at target pressures of approximately 40 mmHg and 25 mmHg at ankle and calf, respectively (Figure 10c).

**Figure 11.**
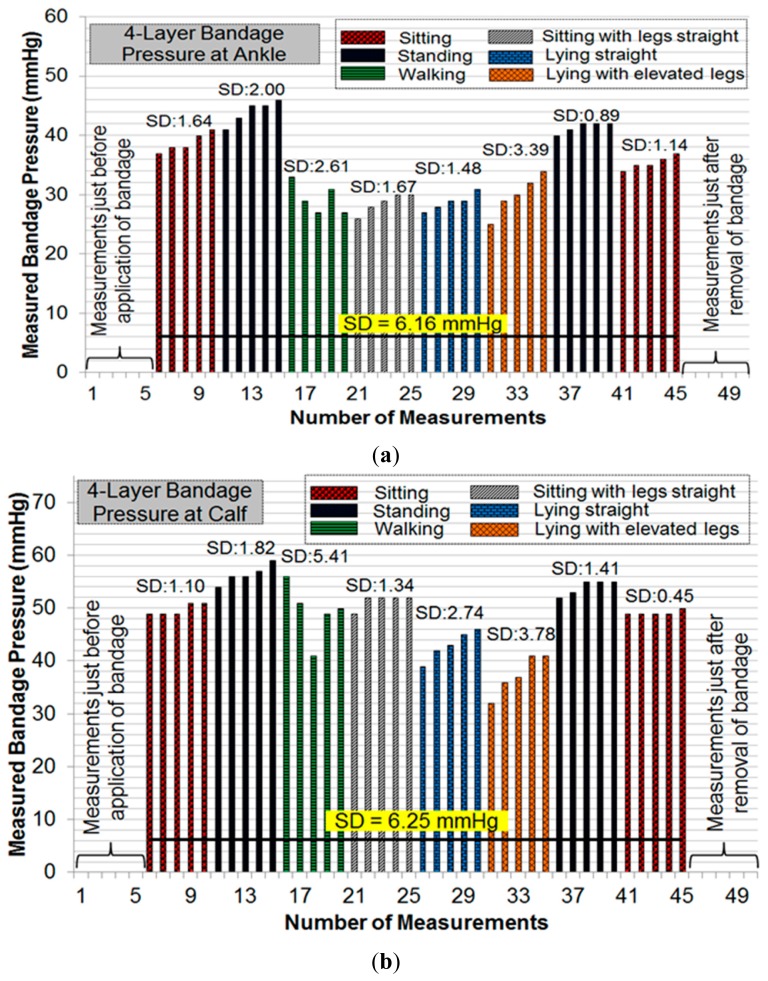
(**a**) Pressure measurements at the ankle with 4-layer bandage; (**b**) pressure measurements at the calf with 4-layer bandage.

**Figure 12.**
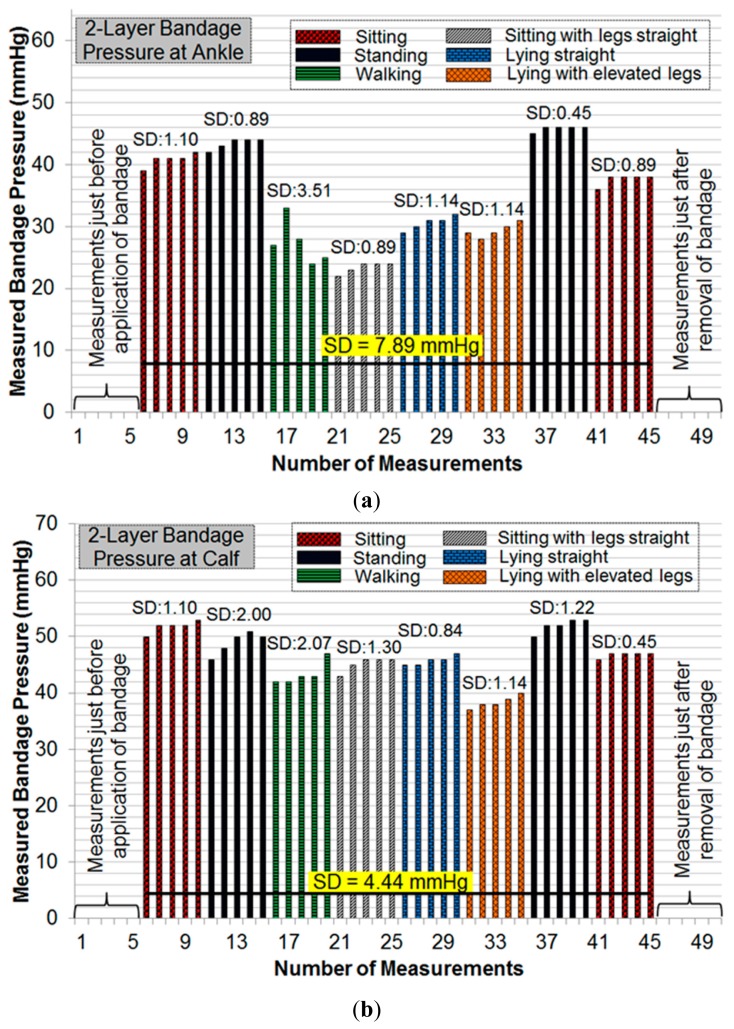
(**a**) Pressure measurements at the ankle with 2-layer bandage; (**b**) pressure measurements at the calf with 2-layer bandage.

Initial pressure readings prior to the application of bandages were recorded to find and nullify any offsets in measurements. Subsequent measurements were taken during common movement postures such as sitting, standing, walking, *etc*. For each posture, five consecutive measurements were recorded. Figure 11 and Figure 12 show the results of pressure measurements using both types of bandages respectively, for all selected body postures. In these figures, the standard deviation (SD) was used as a measure of fluctuation. The SD values for each set-of-five measurements are shown on top of respective bars. The bold horizontal line in each graph indicates the overall fluctuation (SD) with respect to average measured pressure.

**Figure 13.**
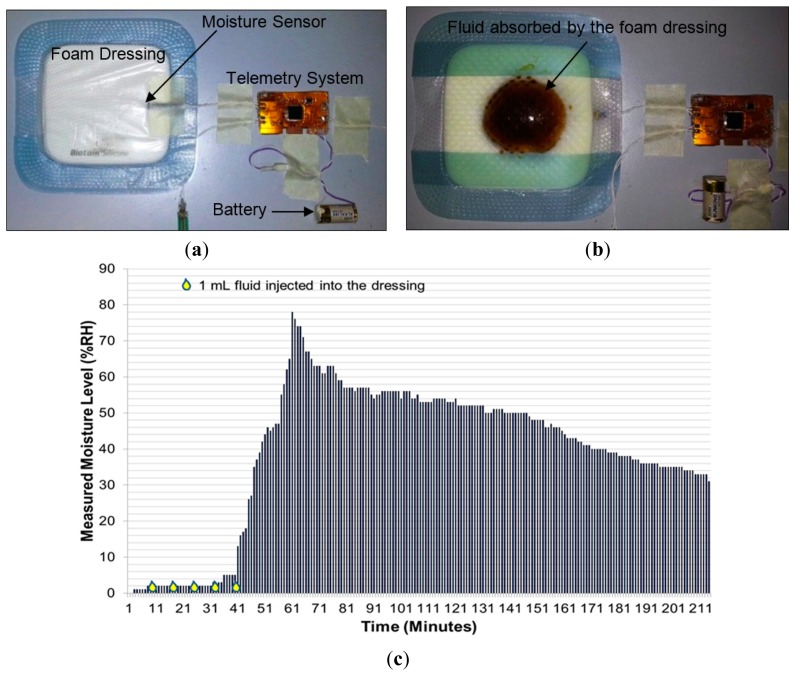
(**a**) Photo of moisture measurement setup showing the moisture sensor inserted into the foam dressing Biatain® Silicone; (**b**) photo showing the spread of injected fluid into the dressing during the experiment; (**c**) graph showing the moisture measurement results during the whole experiment.

Since the moisture measurements do not depend on body movement, a separate test bench was used to validate moisture measurement performance of the sensing system. In this experiment (Figure 13), a 12.5 cm × 12.5 cm moisture-retentive foam dressing (Biatain® Silicone by Coloplast Pty Ltd, Mount Waverley, VIC, Australia) was used. This type of dressing is known to absorb wound exudate and is commonly used for moist-wound healing. A small slit was made to one corner of the dressing and the moisture sensor is placed well inside the foam. The slit was sealed with tape (Figure 13a).

The sensing system was powered using a 6.0 V battery, and initial moisture measurements were recorded on the mobile device. As wound fluid was not available, approximately 10 mL fluid (prepared from black coffee to visualize the spread of fluid in the dressing) was repeatedly injected into the foam dressing until the fluid was observed to reach the vicinity of the moisture sensor (Figure 13b). Measurements are plotted as shown in Figure 13c. This graph showed a higher peak in moisture level as compared to that in Figure 9b, because the fluid volume injected into the foam dressing was greater than that used in the experiment on mannequin leg.

## 5. Discussion

Initial experiments were performed on a mannequin leg to repeat or reconstruct results as and when desired. These results ascertained that the telemetry sensing system is capable of sensing, transmitting, and processing instantaneous changes in the measured parameters with a good degree of accuracy. The algorithm developed for battery voltage-drop compensation played an important role in minimizing measurement errors. The average errors of ±1.91 mmHg and ±0.70 mmHg obtained for high and medium bandage pressures (Figure 9a), respectively, are fit for clinical purpose.

Pressure measurements on a human limb cannot be constant during movement because of many factors involved such as muscle contraction and expansion [12]. We have recorded multiple readings in each movement or posture to isolate the source of fluctuations. Pressure measurements were recorded in a cyclic fashion (sitting→standing→other movements→standing→sitting) to observe whether the measured sub-bandage pressure values return close to the original values obtained earlier for the ‘sitting’ posture. It can be observed from Figures 11 and 12 that this was the case for every experiment. It can also be observed from these measurements that the sensing system reliably measures changes in sub-bandage pressure induced by physical movements. It is worthwhile to mention that the overall fluctuation in sub-bandage pressure is similar for ankle and calf with the 4-layer bandage (around 6 mmHg) whereas for the 2-layer bandage the fluctuation for the calf (4.44 mmHg) is much lower than for the ankle (7.89 mmHg).

As shown in Figure 11a, pressure values for the 4-layer compression bandage at the ankle were relatively stable during ‘sitting’ and ‘standing’ postures (SD: 0.89–2.00 mmHg) as compared to those during ‘walking’ and ‘lying with elevated leg’ (SD: 2.61–3.39 mmHg). This is because the muscle movement is minimal in sitting and standing postures. Similarly, pressure values at the calf were relatively stable during ‘sitting’ and ‘standing’ postures (Figure 11b) (SD: 0.45–1.82 mmHg), while the pressure fluctuated during ‘walking’ and ‘lying straight with elevated leg’ (SD: 3.78–5.41 mmHg). In contrast, for the 2-layer compression bandage, stable ankle pressure values were obtained during all postures, except for ‘walking’ (SD: 2.07 mmHg) as shown in Figure 12a. In the same experiment, the least fluctuations were observed during ‘sitting’ in comparison with those during all other postures. However, the sub-bandage pressure reduced by almost 50% during ‘sitting with legs straight’ in comparison with that obtained during initial ‘sitting’ posture. In Figure 12b, pressure measurements at the calf were relatively stable during all the postures. One common observation from all four experiments in Figures 11 and 12 is that variations in pressure measurements tended to fluctuate during ‘walking’. This is consistent with the variation of physical pressure exerted by muscle contraction and relaxation on the sensor during ‘walking’ [12]. A general observation for postures other than ‘walking’ is that pressure values drifted slightly upwards. For example, sub-bandage pressure drifted from 37 mmHg to 41 mmHg during initial ‘sitting’ posture in Figure 11a. This and similar other drifts might have originated from the phenomenon that muscles took some time to settle on the human limb whenever posture was changed. Until the muscles normalized to that posture, the sensing system was measuring intermediate pressure values. However, no drift in measurements was observed during experiments on mannequin leg mainly because there was no movement.

In Figure 11b and 12b, the average value of measured sub-bandage pressures at calf was around 50 mmHg, while the target value was 25 mmHg. This can be explained by the fact that compression bandaging is designed to move fluid up the leg; therefore a higher pressure is required at the ankle than the calf. The usual values it is aimed to achieve with elastic or multi-layer bandage systems are approximately 40 mmHg at the ankle and 25 mmHg at the calf. However, the Coban™ 2 compression system works quite differently, and these target values are not applicable. Moreover, the sensing system was not aimed at stabilizing the sub-bandage pressure to a target value, but it was instead to measure and display the instantaneous sub-bandage pressure wirelessly.

In summary, the sensing system measured instantaneous variations in sub-bandage pressure with a good level of accuracy and constancy. The pressure sensor, though large in area (38 × 38 mm^2^), is flexible, non-invasive, and adaptable to the limb morphology. Moisture measurements results in Figure 13c also confirmed the reliable and accurate performance of the sensing system in measuring transient changes in moisture level. Initially, when the sensor was dry, the system reported moisture levels less than 2%. Upon injection of external fluid into the foam dressing, the sensor started to show a rise in moisture level as the foam soaked up the moisture. Within a few minutes, the moisture level rose to close to 80% and then gradually decreased as the sensor dried in air. It took more than 100 min for the moisture to drop from 80% to 40% since the dressing used in this experiment was a moisture-retentive dressing designed to hold moisture over a long period.

## 6. Conclusions

In this paper, we have presented a real-time, flexible, and mobile-based sensing and monitoring system for chronic wound monitoring applications. The system has advanced features and improved performance, and is capable of monitoring pressure at two different locations on a human limb such as above the ankle and on the calf muscle. Similarly, one moisture sensor could serve as a reference sensor placed over the skin while the other one measures the moisture level of wound fluid through a moist dressing. The size of the system was suitable for placement under a compression bandage on a human limb. The sensing system reliably communicated with a mobile device through a matched receiver, up to a distance of 5 m. A custom-designed Android application received, processed, and displayed the received parameters' values in real-time, in text and graphical formats. The system automatically acquired data at a determined interval of 5 s and saved it to a file with time stamps for analysis by the clinician. The system ran on an external battery which discharged over time, thus gradually degrading the sensors' measurement capability. A compensation algorithm based on first degree mathematical polynomial was designed and implemented to overcome this degradation to a large extent.

Experiments on a mannequin leg ascertained the reliable and repeatable performance of the monitoring system. Further experiments on a healthy human volunteer using commonly-used compression bandages showed that the system reliably and non-invasively measures transient changes in bandage pressure instantly as they arise from normal movements. Experiments with a moisture-retentive foam dressing have also proven reliable and provided consistent moisture measurements. Future work will include trials of the improved sensing system on a cohort of healthy human volunteers. The device will also be tested on patients with venous leg ulcers after improvements and due ethical approvals. To the best of our knowledge, the developed device is a pioneer effort in practically utilizing the sensing and wireless technologies for chronic wound diagnostics. As the sensing and bio-sensing technologies are maturing, it is anticipated that with suitable sensors and interface electronics, the proposed technology could potentially be used for other wireless biomedical applications beyond the scope of wound monitoring, e.g., heart beat monitoring for heart patients, joint monitoring for fractured bones *etc*. This technology may ultimately revolutionize the way the chronic wounds are treated and monitored. It will potentially lead to synthesize the futuristic dressings that could sense, think, heal and report autonomously.
